# Long-term survival outcomes of Wagner™ conical stems in crowe non-IV hip dysplasia: a retrospective analysis

**DOI:** 10.3389/fsurg.2025.1701518

**Published:** 2025-10-24

**Authors:** Fabio D’Angelo, Andrea Pautasso, Delia Antognazza, Luca Monestier, Mattia Gervasini, Marco Filipponi, Chiara Bernardi, Giacomo Riva

**Affiliations:** 1 Orthopaedic and Traumatology Department, ASST Sette Laghi—Circolo Hospital and Macchi Foundation in Varese—University Hospital, Varese, Italy; 2 Department of Biotechnology and Life Sciences (DBSV), University of Studies Insubria, Varese, Italy; 3 University of studies Insubria, Varese, Italy; 4 Orthopaedic and Traumatology Department, Ospedale Vito Fazzi, Lecce, Italy

**Keywords:** hip dysplasia development, Wagner stem, total hip arthroplasty, orthopedic implants survival, THA complication

## Abstract

**Background:**

Total hip arthroplasty (THA) is one of the most frequently performed orthopedic procedures. Developmental dysplasia of the hip (DDH) presents specific anatomical challenges that require tailored implant designs. Wagner™ conical stems were developed to address the morphological alterations of dysplastic femur, offering potential advantages in stability and functional restoration. This study assesses the long-term survival and clinical outcomes of Wagner™ conical stems in patients with Crowe non-IV DDH.

**Materials and methods:**

This retrospective study included primary THAs performed between 2003 and 2015 using Wagner™ conical stems exclusively in patients with DDH. Only cases with complete clinical and radiographic follow-up were analyzed, excluding those lost to follow-up or revised. The evaluated outcomes were prosthetic survival rate, clinical performance assessed with the Modified Harris Hip Score (mHHS), and radiographic findings at final follow-up.

**Results:**

Forty-five patients (57 hips) met the inclusion criteria. The mean age at surgery was 56.5 years (range, 33–76), with a mean follow-up of 15 years (range, 8–20). Kaplan–Meier analysis showed a survival rate of 95.7% at 10 years and approximately 80.5% at 20 years, with an overall survival rate of 89.5% at the final follow-up. The main cause of failure was aseptic loosening, followed by infection, recurrent dislocation, and metallosis. Among hips with retained implants, 86.3% (44/51) achieved an mHHS > 70, indicating satisfactory functional recovery. The mean mHHS was 88.6 ± 14.3 (Range, 62–100), with a median of 92 and a mode of 100.

**Conclusion:**

Wagner™ conical stems provide durable fixation and favorable long-term functional outcomes in Crowe non-IV DDH, with high survival rates and low complication incidence over extended follow-up.

## 1 Introduction

Developmental dysplasia of the hip (DDH) represents a continuum of structural abnormalities involving the acetabulum and proximal femur, ranging from mild acetabular shallowing to complete femoral head dislocation. These deformities disrupt the concentric congruency of the hip joint and impair the physiological distribution of load across articular surfaces, producing altered biomechanics that predispose to early degenerative changes. In untreated cases, the abnormal stress concentration accelerates cartilage wear, leading to pain, functional impairment, and often premature osteoarthritis (OA) [1].

The reported incidence of DDH varies widely, from 1 to 34 per 1,000 live births, reflecting differences in geographic region, ethnicity, screening protocols, and diagnostic criteria [2]. Higher prevalence has been observed in populations practicing traditional tight swaddling and in those with familial predisposition [3]. Female sex is a strong risk factor, with a female-to-male ratio of approximately 6:1. The left hip is more frequently affected than the right, likely due to intrauterine positioning with the left hip adducted against the maternal lumbosacral spine [4].

If DDH is not detected and treated in infancy, residual dysplasia can persist into adolescence and adulthood. This residual deformity is a major predisposing factor for early-onset hip OA, often presenting in the third or fourth decade of life. Epidemiological studies have estimated that untreated or inadequately treated DDH accounts for up to 20%–40% of total hip arthroplasties (THAs) performed in patients younger than 50 years [1, 5].

Accurate classification of DDH severity is essential for surgical planning, particularly in adult THA. The Crowe classification is among the most widely used systems, grading severity based on the percentage of proximal displacement of the femoral head relative to the height of the pelvis [5]. Four categories are defined: Crowe I (<50% displacement), Crowe II (50%–75%), Crowe III (75%–100%), and Crowe IV (>100% displacement). While useful for assessing femoral head displacement, it provides limited information on acetabular morphology. The Hartofilakidis classification combines radiographic and anatomical considerations, describing dysplasia (femoral head contained in a shallow true acetabulum), low dislocation (partial articulation with a false acetabulum while retaining some contact with the true acetabulum), and high dislocation (femoral head completely outside the true acetabulum) [6, 7].

Both classifications are valid and reliable [8–10]. Table 1 shows a comparison between the Crowe and Hartofilakid systems.

**Table 1:** Crowe vs. Hartofilakidis classification.

Parameters	Crowe classification	Hartofilakidis classification
Focus	Vertical displacement of the femoral head	Morphological features of the acetabulum and femoral head
Basis	Radiographic measurement of subluxation/dislocation (% of pelvis height)	Anatomical relationship and shape of acetabulum
Used in	Preoperative planning for THA	Surgical planning and prognosis in adult dysplasia
Number of types	4 (Type I–IV)	3 (Dysplasia, low dislocation, high dislocation)
Advantages	Simple, quantitative, reproducible	Morphological, helps understand acetabular anatomy
Limitations	Does not assess acetabular shape or quality	Less quantitative, more subjective

More recently, Wells et al. performed a detailed three-dimensional CT analysis of hip morphology in adult DDH [11]. Their work highlighted the complex structural variations of the acetabulum and proximal femur in this population, demonstrating a high prevalence of cam-type deformities and reduced head–neck offset. Such morphologic characterization can assist in preoperative planning by identifying potential femoral head–neck abnormalities that may require corrective osteochondroplasty, and by providing a more comprehensive understanding of the anatomic challenges relevant to implant positioning and joint biomechanics in THA.

Performing THA in the setting of DDH presents unique technical challenges. On the acetabular side, the socket is often shallow, dysplastic, and anteverted, with deficient bone stock that may require structural grafting, augments, or custom implants. On the femoral side, the canal is frequently narrow, stovepipe-shaped, and excessively anteverted, with altered metaphyseal flare and offset [12]. These morphological characteristics complicate stem insertion, increase the risk of cortical perforation or malalignment, and may compromise primary stability.

To address these challenges, specific femoral stem designs have been developed. Among them, conical stems with longitudinal ribs can achieve diaphyseal fixation and provide axial and rotational stability, even in narrow or distorted canals [13, 14]. While short- to mid-term results of such stems in DDH have been encouraging, with high rates of osseointegration and low mechanical failure [15, 16], data on long-term survival, functional performance, and complication rates—particularly in Crowe non-IV hips—remain limited.

The present study aims to evaluate the long-term survival, clinical outcomes, and radiographic performance of Wagner™ conical stems in patients with Crowe non-IV DDH, thereby contributing evidence to guide implant selection in complex primary THA.

## 2 Materials and methods

### 2.1 Study sample

This observational retrospective cohort study included all patients who underwent total hip arthroplasty (THA) between January 2003 and December 2015 for Crowe type I–III developmental dysplasia of the hip (DDH) using a Wagner™ conical stem at our institute (ASST Sette Laghi—Circolo Hospital and Macchi Foundation, University Hospital of Varese, Italy).

Inclusion criteria were survival to the most recent follow-up and absence of any revision procedure. Patients were excluded if they had undergone revision surgery for any reason between the index operation and the last follow-up, or if they had neurological disorders that could compromise functional assessment; these cases were nevertheless analyzed separately to document complications and identify the causes of failure or revision.

### 2.2 Surgical technique and prosthesis

All procedures were performed through a posterolateral hip approach. Following femoral head dislocation, a neck osteotomy was executed at the level determined during preoperative templating to ensure adequate acetabular exposure. The acetabular component was implanted first, with dedicated reamers used to achieve the planned center of rotation and correct inclination and anteversion.

Preoperative planning was considered critical, given the altered femoroacetabular morphology in DDH, which may compromise implant positioning and long-term stability. To allow neutral access for sequential conical reamers, bone was excised from the medial greater trochanter and lateral femoral neck. Reaming progressed incrementally until uniform circumferential cortical contact was achieved, establishing the distal trial diameter.

A modular trial stem, matching the final reamer size and selected neck angle, was inserted to assess femoral anteversion, offset, leg length, and range of motion with a provisional head. Once the desired biomechanics were confirmed, the definitive stem was impacted to the planned depth and orientation using the dedicated impactor. The femoral head was mounted on the 12/14 taper with controlled axial impaction, and a final reduction was performed to confirm joint stability, limb length equality, and appropriate soft-tissue tension before closure.

The Wagner Cone Prosthesis (Zimmer Biomet, Warsaw, IN, USA) has a $5^{\circ }$ conical taper and a circular cross-section, with eight sharp longitudinal ribs to enhance axial and rotational stability through cortical engagement. Two neck-shaft angles are available ($125^{\circ }$ and $135^{\circ }$) to facilitate restoration of femoral offset and limb length, while the range of twelve stem diameters and modular components provides intraoperative flexibility for sizing and adjusting anteversion [14].

### 2.3 Patient assessment at follow-up

All patients were evaluated clinically and radiographically at follow-up by the same surgeon (DA). In cases where the primary implant was retained (no revision or removal by 31 December 2023), the Modified Harris Hip Score (mHHS) was administered.

The mHHS assesses both pain and function: the pain domain assigns up to 44 points for absence of pain, with progressively lower scores for increasing severity; the function domain, with a maximum of 47 points, evaluates limp, use of walking aids, walking distance, ability to climb stairs, to put on socks and shoes, to sit for prolonged periods, and to use public transport. The total score is multiplied by 1.1 to yield a range from 0 to 100. Based on the original Harris Hip Score categories, scores are classified as excellent (90–100), good (80–89), fair (70–79), or poor (<70).

Radiographs were reviewed for complications, with implant loosening defined as subsidence greater than 2 mm. Femoral stem axial subsidence was measured on standardized anteroposterior pelvis and hip radiographs as the change in vertical distance from the prosthetic shoulder to the tip of the greater trochanter between serial examinations.

For patients who underwent revision, the time from the primary surgery and the reason for revision were recorded.

### 2.4 Statistical analysis

Data were analyzed using IBM SPSS Statistics software (IBM Corp., Armonk, NY, USA). Qualitative variables, such as sex, were described using frequency distribution, mode, Gini index, and normalized Gini index; quantitative variables, including patient age at surgery and mHHS scores, were summarized as mean, median, and variance. Kaplan–Meier survival analysis was used to assess implant survivorship, with revision for any reason as the endpoint. No imputation was performed for missing data, and all analyses were conducted using available cases. The threshold for statistical significance was set at *p* < 0.01.

## 3 Results

During the study period, 121 primary total hip arthroplasties (THAs) were performed using a Wagner™ type conical stem. In all cases, the indication was secondary osteoarthritis due to developmental dysplasia of the hip (DDH). These procedures were carried out in 103 patients, 18 of whom underwent bilateral surgery.

**Figure 1:**
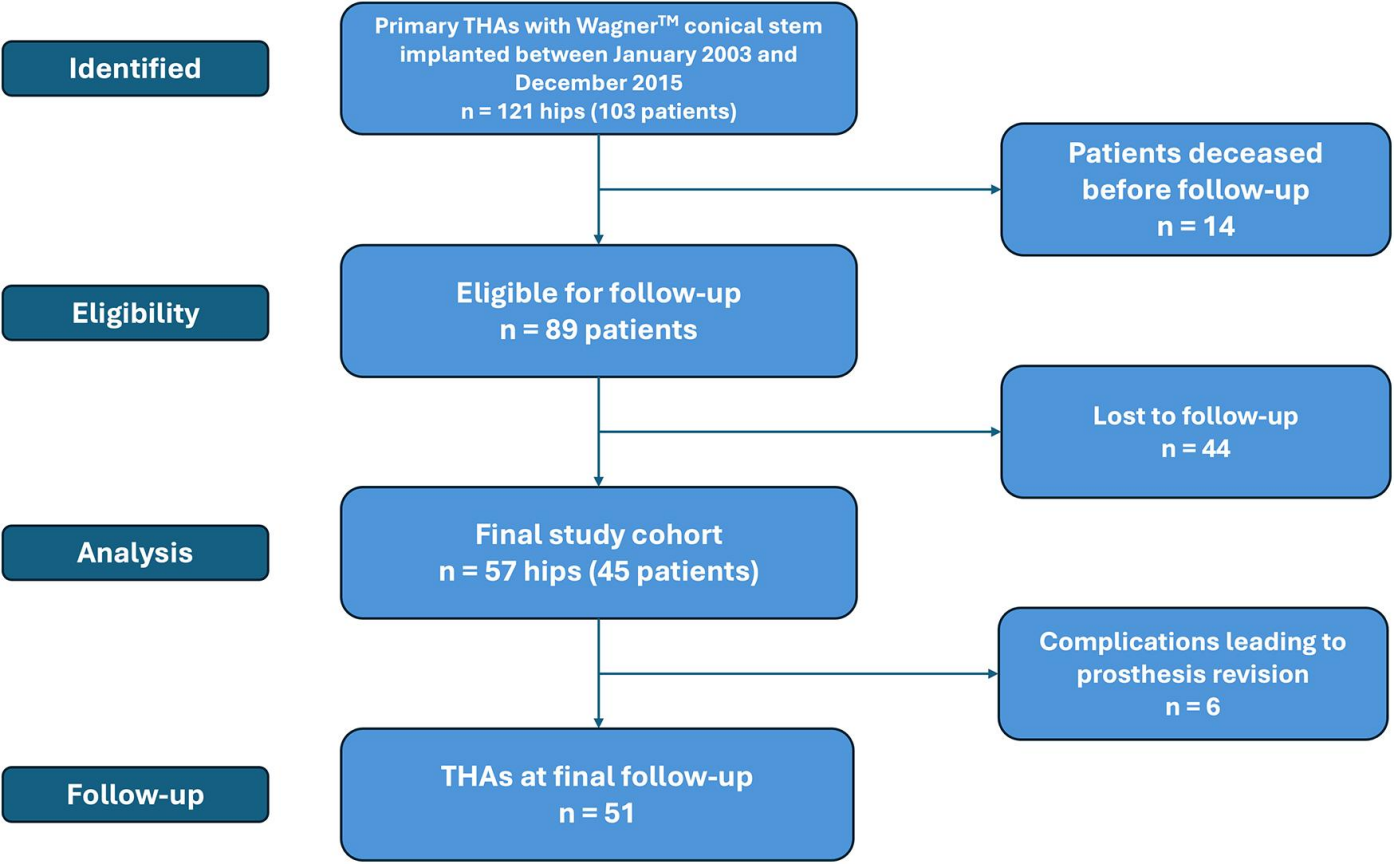
CONSORT flow-diagram for patients who underwent total hip arthroplasty with a Wagner™ conical stem.

Among the 103 patients, 14 died before follow-up contact and 44—including 6 with bilateral procedures—were lost to follow-up. The final study cohort comprised 45 patients, 12 of whom (26.7%) underwent bilateral arthroplasty, resulting in 57 hips included in the analysis (Figure 1). The mean follow-up duration was 15.4 ± 3.8 years (range, 8–20). The cohort included 6 men (13.3%) and 39 women (86.7%), with a mean age at surgery of 56.5 ± 10.8 years (range, 33–76).

**Figure 2:**
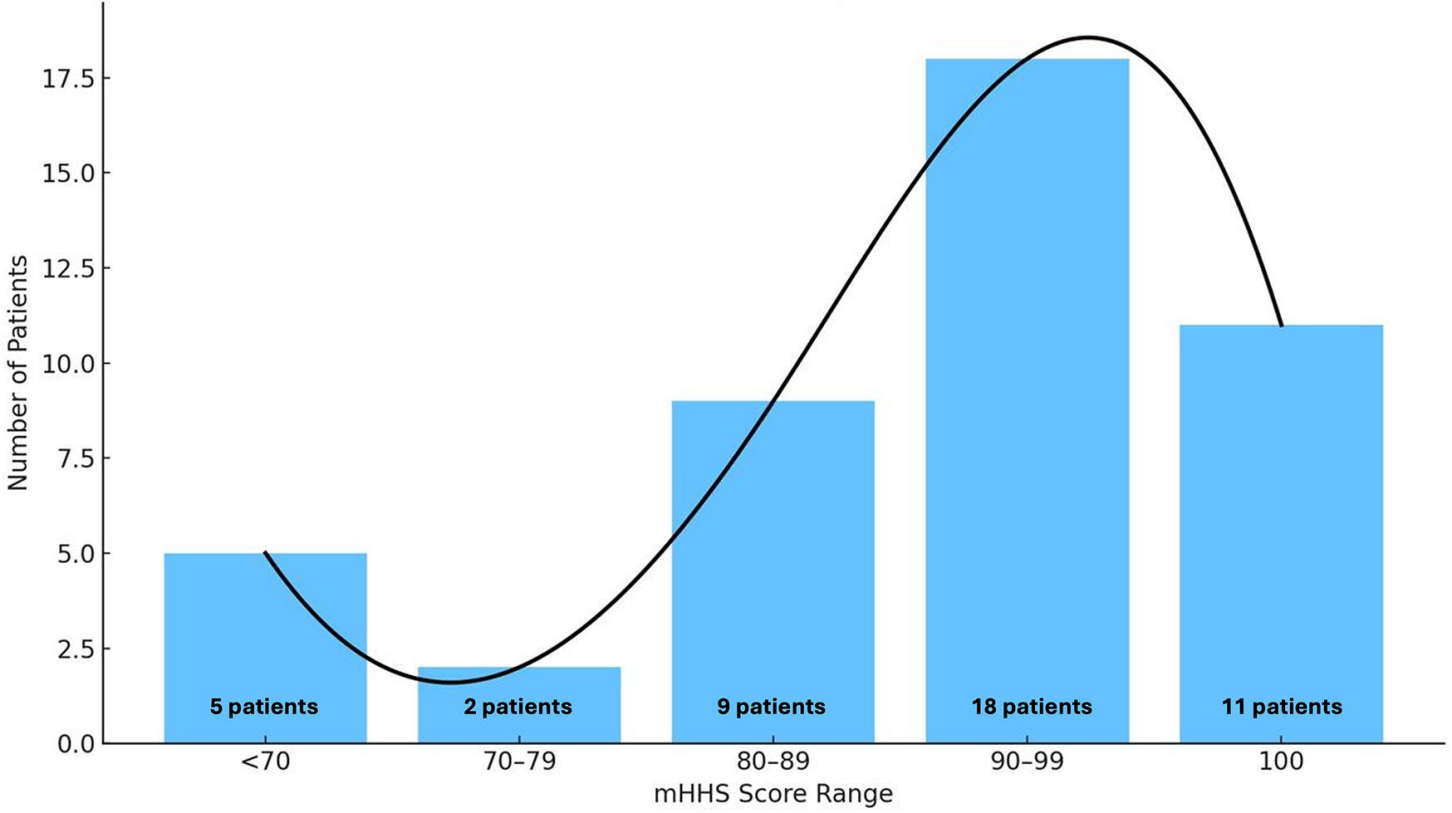
Absolute frequency of mHHS scores in the group under examination.

At the latest clinical assessment (December 31, 2023), among patients who retained the original implant, 44 out of 51 hips (86.3%) achieved a Modified Harris Hip Score (mHHS) above 70—the threshold below which hip function is considered poor. The mean mHHS was 88.6 ± 14.3 (Range, 62–100), with a median of 92 and a mode of 100, reflecting a marked right-skewed distribution (Figure 2).

The most frequently implanted stem diameter was 17 mm, used in 15 hips (26.3%). The most common acetabular component size was 48 mm, employed in 21 cases (36.8%).

Implant failure occurred in 6 hips (10.5%), with a mean time to failure of 9.2 ± 6.2 years (range, 0–19) and a median of 9.5 years. The leading cause was aseptic loosening, accounting for 3 cases (50% of failures): one involved the femoral stem at 10 years postoperatively, and two involved the acetabular component—one at 9 years and one within 12 days of surgery. Other causes included deep infection at 6 years (*n* = 1), recurrent dislocations at 11 years (*n* = 1), and metallosis with pseudotumor formation, painful gait, and elevated serum chromium levels at 15 years (*n* = 1). Table 2 summarizes the causes of implant failure and the timing of revision surgery.

**Table 2:** Causes of failure/revision and time to revision in the study cohort.

Cause of failure/revision	Number of cases (*n*)	% of failures	% of total hips (*n* = 57)	Time to revision (years)
Aseptic loosening—femoral component	1	16.7%	1.8%	10
Aseptic loosening—acetabular component	2	33.3%	3.5%	0.03 and 9
Deep infection	1	16.7%	1.8%	6
Recurrent dislocation	1	16.7%	1.8%	11
Metallosis with pseudotumor	1	16.7%	1.8%	15
Total	6	100%	10.5%	Mean: 9.2 (range 0–19)

Radiographic follow-up identified two cases of femoral stem subsidence greater than 2 mm, both in asymptomatic patients with optimal mHHS scores. No radiographic evidence of progressive osteolysis, component migration, or heterotopic ossification (Brooker grade ≥ II) was observed in hips with retained implants.

**Figure 3:**
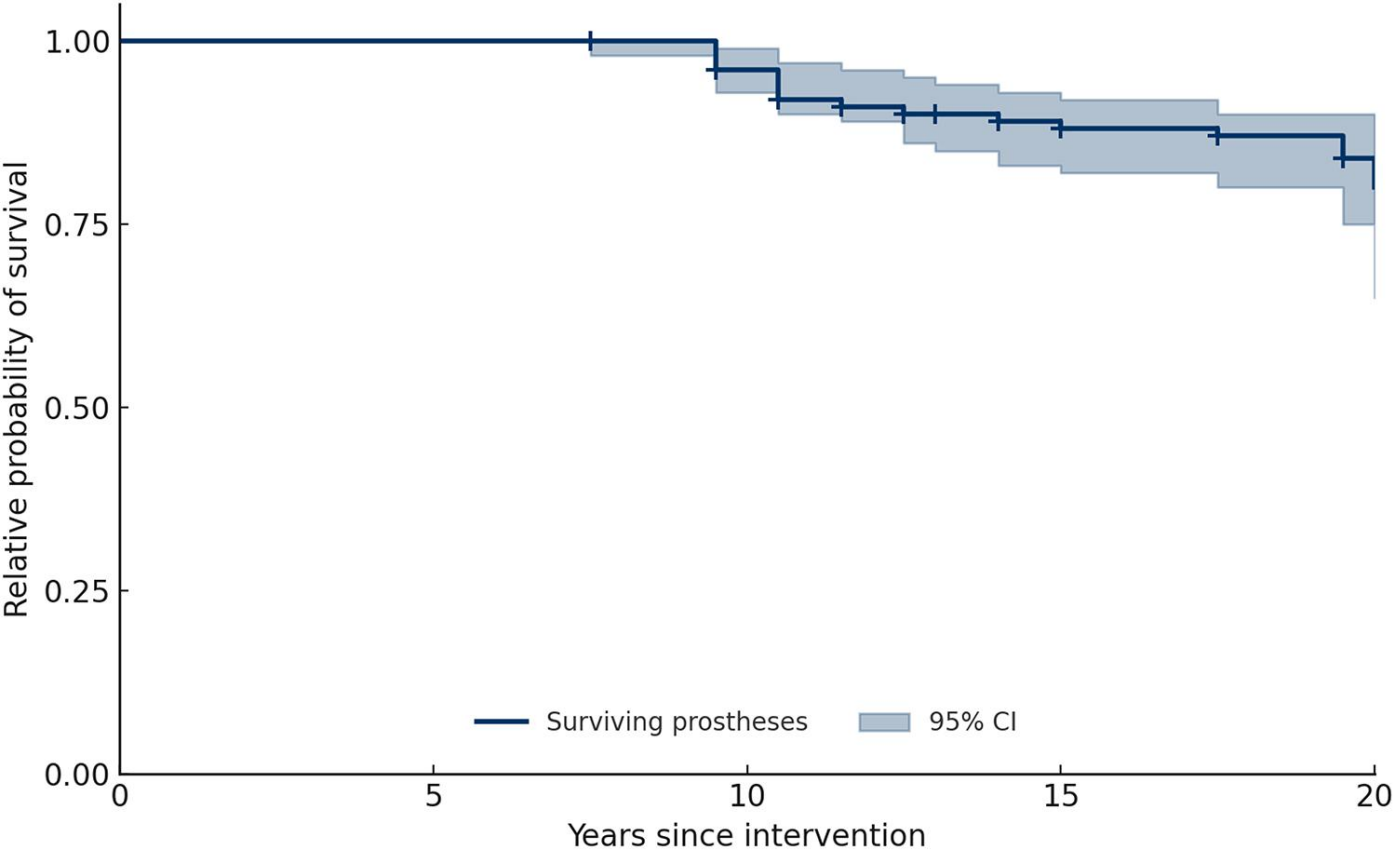
Kaplan–Meier survival curve of Wagner™ conical stems over 20 years.

Kaplan–Meier survival analysis demonstrated cumulative survival probabilities of 95.7% at 10 years and 80.5% at 20 years, with an overall survival rate of 89.5% at the final follow-up (Figure 3).

## 4 Discussion

Developmental dysplasia of the hip (DDH) encompasses a wide spectrum of structural abnormalities involving the acetabulum and proximal femur, each of which poses unique challenges in the setting of total hip arthroplasty (THA) [2, 4]. Untreated DDH can lead to early-onset osteoarthritis, frequently requiring THA in young to middle-aged adults [2, 4]. In this population, the anatomy of the femur often presents a narrow diaphyseal canal, increased anteversion, and altered metaphyseal flare [12], which make implantation of standard metaphyseal-fitting stems technically demanding and sometimes biomechanically suboptimal. The choice of femoral stem is therefore critical to achieve long-term stability, restore biomechanics, and reduce the risk of complications.

Conical stems with distal fixation, such as the Wagner™ design, were originally developed for revision arthroplasty but have since been adopted for complex primary THA, including DDH, due to their ability to bypass the distorted metaphyseal anatomy and engage the more uniform diaphyseal cortical bone [14]. Compared with cylindrical diaphyseal stems, conical designs offer the advantage of gradual axial load transfer, potentially reducing stress shielding and facilitating stable osseointegration (14, 17). In contrast, fully modular stems may allow for more precise version correction but carry the risk of mechanical junctional failure or corrosion [18].

**Figure 4:**
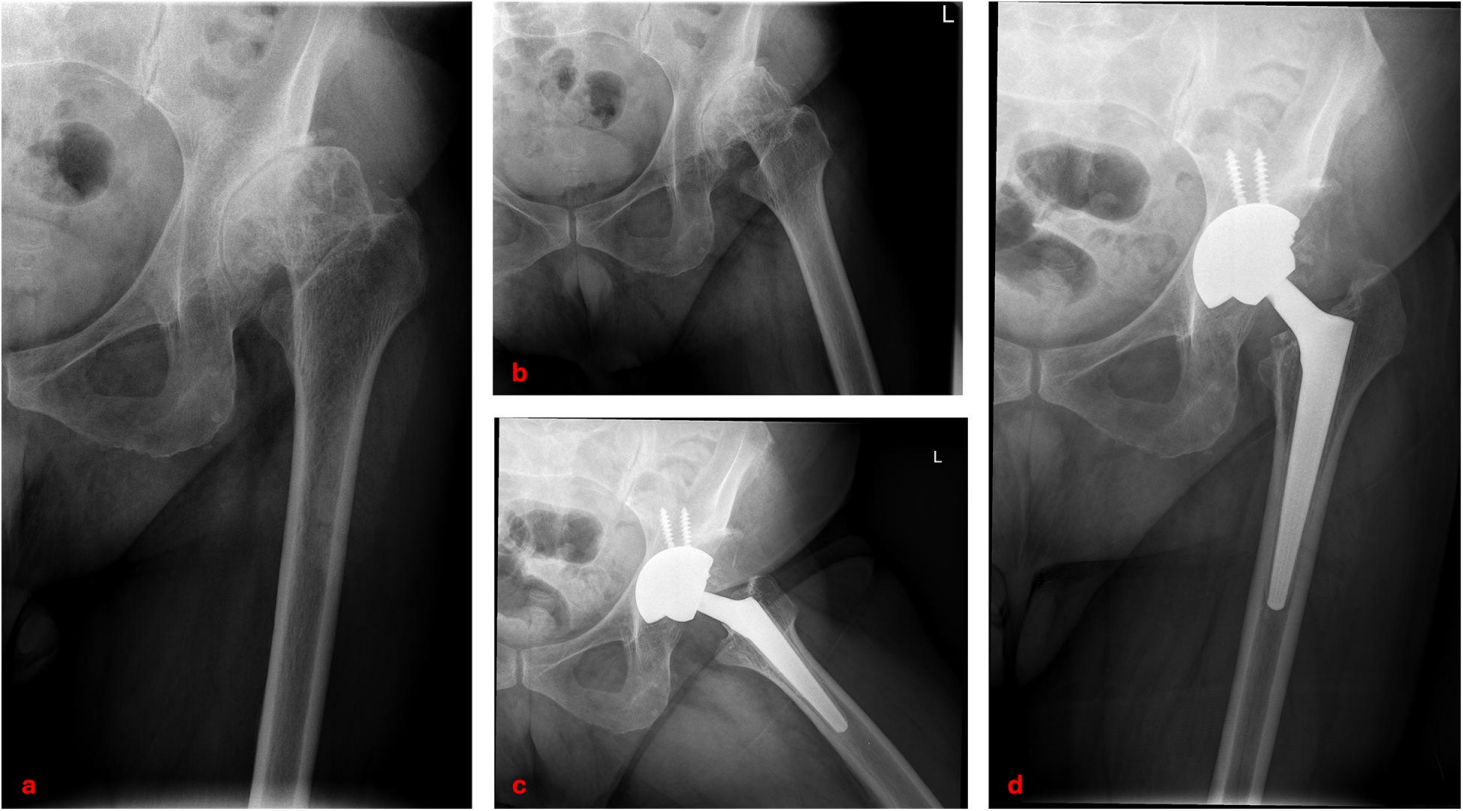
Clinical case of left hip developmental dysplasia (Crowe type III): preoperative anteroposterior and lateral radiographs **(a,b)**; postoperative radiographs at 14-year follow-up showing total hip arthroplasty with Wagner™ conical stem **(c,d)**.

The aim of the present study was to evaluate the long-term survival, clinical outcomes, and radiographic performance of Wagner™ conical stems in patients with Crowe non-IV DDH undergoing primary THA (Figure 4).

Our findings demonstrated excellent long-term survivorship, with Kaplan–Meier estimates of 95.7% at 10 years and 80.5% at 20 years. These results are consistent with previous series using similar stems in DDH. Solarino et al. [13] reported survival rates of 99.2% (95% CI 92.8–100) at 5 years, 95.1% (95% CI 87.5–98.9) at 10 years, and 81.3% (95% CI 88.7–66.4) at 20 years in a mixed Crowe I–IV population, while Fahlbusch et al. [19] documented high survivorship beyond 21 years, with 98.3% survival at 10 years and 81.8% at final follow-up. Functional outcomes in our cohort were also favorable, with a mean mHHS of 88.6 and 86.3% of hips scoring above 70 points, indicating satisfactory recovery. This agrees with Faldini et al. [20], who found a mean Harris Hip Score of 90 ± 9 (range 81–100) at 12 months after surgery, and 91 ± 8 (range 83–100) at last follow-up in Crowe II–III hips, and with Grappiolo et al. [21], who reported mean scores of 90.3 (range, 62–100) at last follow-up (*p* < 0.001) in Crowe IV hips treated with monoblock conical stems and shortening osteotomy, with a survivorship of 95.9% (95%IC, 91.9%–99.9%) at ten years.

Radiographically, 94.4% of stems achieved stable osseointegration without signs of loosening, with only two cases of subsidence >2 mm, both asymptomatic and associated with excellent clinical scores. This low subsidence rate is comparable to that reported by Zhen et al. [16] (3.9%), and supports the role of distal fixation in ensuring long-term stability in dysplastic femurs.

**Figure 5:**
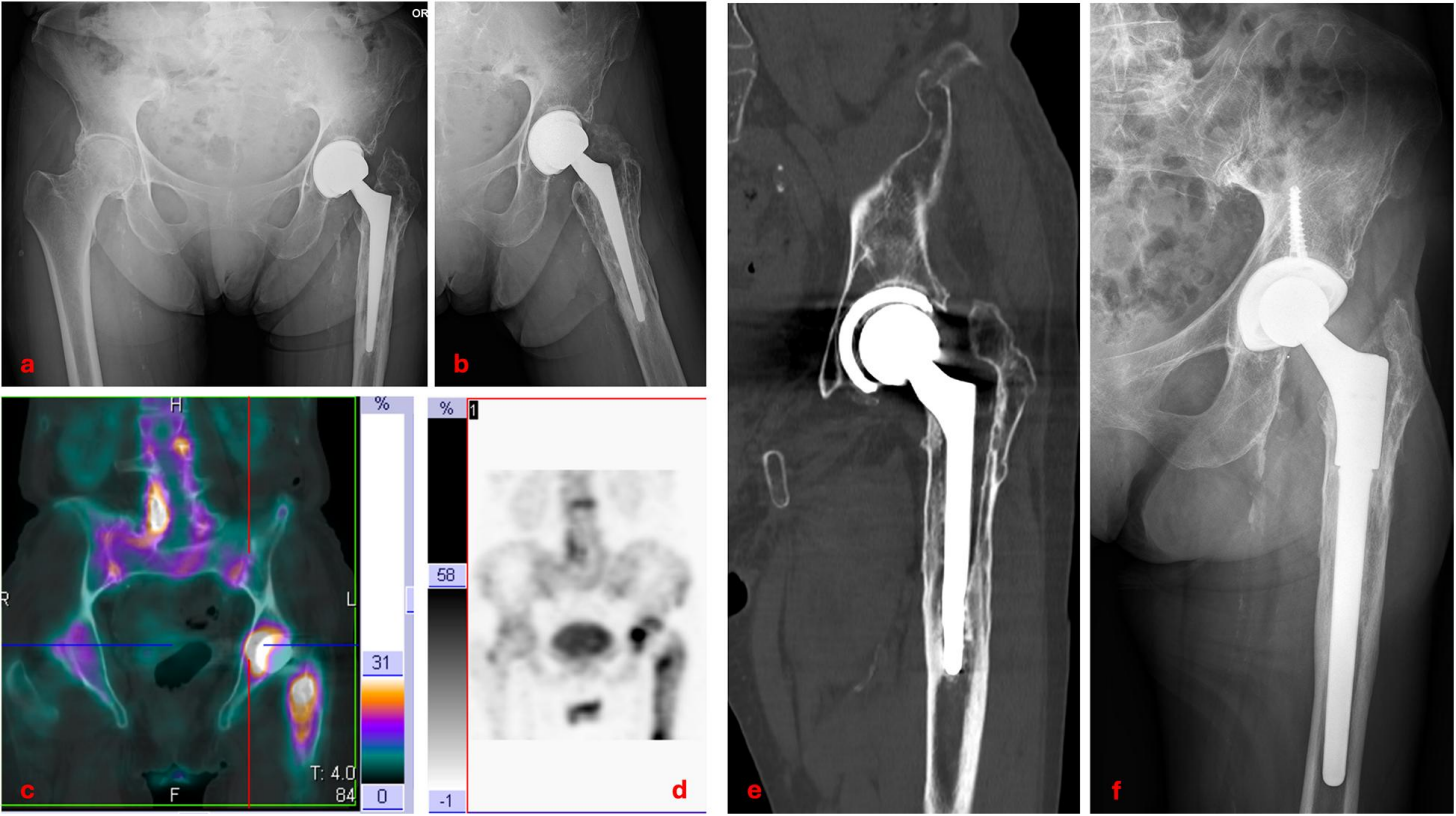
Clinical case of aseptic loosening of total hip arthroplasty: anteroposterior and lateral radiographs **(a,b)**; baseline three-phase bone scintigraphy with 99mTc showing increased uptake around the prosthesis **(c,d)**; coronal CT scan demonstrating loosening of both the femoral stem and acetabular cup **(e)**; postoperative radiograph after revision surgery with a new modular stem **(f)**.

The complication profile in our series was low, with an overall failure rate of 10.5% over nearly two decades. Aseptic loosening (Figure 5), the most frequent cause of revision in DDH THA, occurred in three cases (5.2%), a rate that falls within the 2%–10% range reported in long-term series [13, 16, 20]. Notably, one acetabular component failed within 12 days postoperatively, suggesting primary fixation problems rather than biological failure. Deep infection occurred in one case (1.8%), which is consistent with the 0.5%–2% incidence generally reported for primary THA [22]. In the literature, dislocation rates for THA in DDH vary widely: Mortazavi et al. [23] found a rate of 12% in a series of 171 patients, while Ding et al. [24] documented dislocation in 22 of 524 hips (4.2%) treated for dysplasia. The dislocation rate in our study was 1.8%, which may reflect meticulous preoperative planning and careful restoration of offset, version, and soft-tissue tension. The single case of metallosis, presenting with pseudotumor formation 15 years after surgery, highlights the importance of long-term follow-up, especially where metal debris from modular junctions may accumulate over time.

Meticulous preoperative planning was critical to these outcomes. In DDH, both acetabular and femoral deformities must be addressed to optimize implant position and biomechanics. 3D CT studies, such as that by Wells et al. [11], have demonstrated that cam-type deformities and reduced head–neck offset are common in DDH, potentially limiting range of motion and predisposing to impingement. In our series, controlled bone removal from the greater trochanter and lateral femoral neck was key to achieving a neutral reaming trajectory, minimizing malalignment risk and maximizing stem stability.

From a clinical perspective, the high survivorship and functional recovery observed support the use of conical stems in Crowe non-IV DDH, particularly in younger patients for whom implant longevity is essential. The modularity of the Wagner™ system allows intraoperative adjustments in leg length and offset, which are crucial for restoring normal gait mechanics and preventing long-term complications such as abductor insufficiency or pelvic obliquity.

This study has several limitations. First, its retrospective design is inherently subject to selection bias. Second, the relatively high proportion of patients lost to follow-up (43%) may reduce the precision of survival estimates and potentially lead to an overestimation of implant survivorship, as failures occurring outside our institution could not be systematically captured. In fact, considering the long follow-up interval of 15–20 years, many patients were elderly at the time of review and were unable to attend new clinical or radiographic assessments due to significant comorbidities or relocation to different geographical areas. In several cases, clinical information was also unavailable because patients had transitioned to nursing facilities or local healthcare services, limiting our ability to recontact them. These factors are common challenges in long-term arthroplasty studies and reflect the inherent difficulty of maintaining complete cohorts over decades. Further limitations are the absence of a control group treated with alternative stem designs, which prevents direct comparative conclusions, and the reliance on plain radiographs for radiographic evaluation, which may underestimate subtle osteolysis or early implant migration.

Despite these limitations, this study provides a long-term follow-up series of Wagner™ stems in Crowe non-IV DDH, showing high survivorship, excellent functional outcomes, and a low complication rate over nearly two decades. These results contribute to the growing evidence supporting conical stems as a reliable solution for complex primary THA in dysplastic hips.

## 5 Conclusions

This long-term follow-up study demonstrates that Wagner™-type conical stems provide excellent survivorship, satisfactory functional recovery, and a low complication rate in patients with Crowe non-IV DDH undergoing primary THA. These results, consistent with the most recent literature, support conical stems as a reliable option for managing the complex femoral anatomy typically associated with dysplasia. Careful preoperative planning, accurate correction of deformities, and appropriate intraoperative adjustments remain essential to optimizing implant positioning, restoring biomechanics, and maximizing long-term outcomes.

## Data Availability

The original contributions presented in the study are included in the article/Supplementary Material, further inquiries can be directed to the corresponding author.
